# Artificial Shape Perception Retina Network Based on Tunable Memristive Neurons

**DOI:** 10.1038/s41598-018-31958-6

**Published:** 2018-09-13

**Authors:** Lin Bao, Jian Kang, Yichen Fang, Zhizhen Yu, Zongwei Wang, Yuchao Yang, Yimao Cai, Ru Huang

**Affiliations:** 1 Institute of Microelectronics, Peking University, Beijing, 100871 China; 2 Innovation Centre for Microelectronics and Integrated System, Peking University, Beijing, 100871 China

## Abstract

Retina shows an extremely high signal processing efficiency because of its specific signal processing strategy which called computing in sensor. In retina, photoreceptor cells encode light signals into spikes and ganglion cells finish the shape perception process. In order to realize the neuromorphic vision sensor, the one-transistor-one-memristor (1T1M) structure which formed by one memristor and one MOSFET in serial is used to construct photoreceptor cell and ganglion cell. The voltage changes between two terminals of memristor and MOSFET can mimic the changes of membrane potential caused by spikes and illumination respectively. In this paper, the tunable memristive neurons with 1T1M structures are built. According to the concept of receptive field of ganglion cells (GCs) in the retina, the artificial shape perception retina network is constructed with these memristive neurons. The final results show that the artificial retina can extract shape information from the image and transfer it into spike frequency realizing the function of computing in sensor.

## Introduction

It makes a great sense for using biological principles to design microelectronic circuits^1,2^. On one hand, neural system has many hierarchical and parallel signal processing units. In the retina, every photoreceptor cell is a processing unit which encode illumination signal into spikes. Each ganglion cell connects with several photoreceptor cells and detects the activity differences between these cells. Because of this highly parallel computing strategy, it only takes two steps for retina to finish shape perception process which shows much higher efficiency than traditional circuits. On the other hand, retina is not only a perceptive organ but an important component of central nervous system which means that the information can be processed in the retina. In other words, the retina shows a specific computing strategy which called computing in sensor. This locally computing strategy reduces the transmission cost between sensors and processors, so that the signal processing can be faster. So, the artificial retina network will be faster and smarter than traditional image processing device.

**Figure 1:**
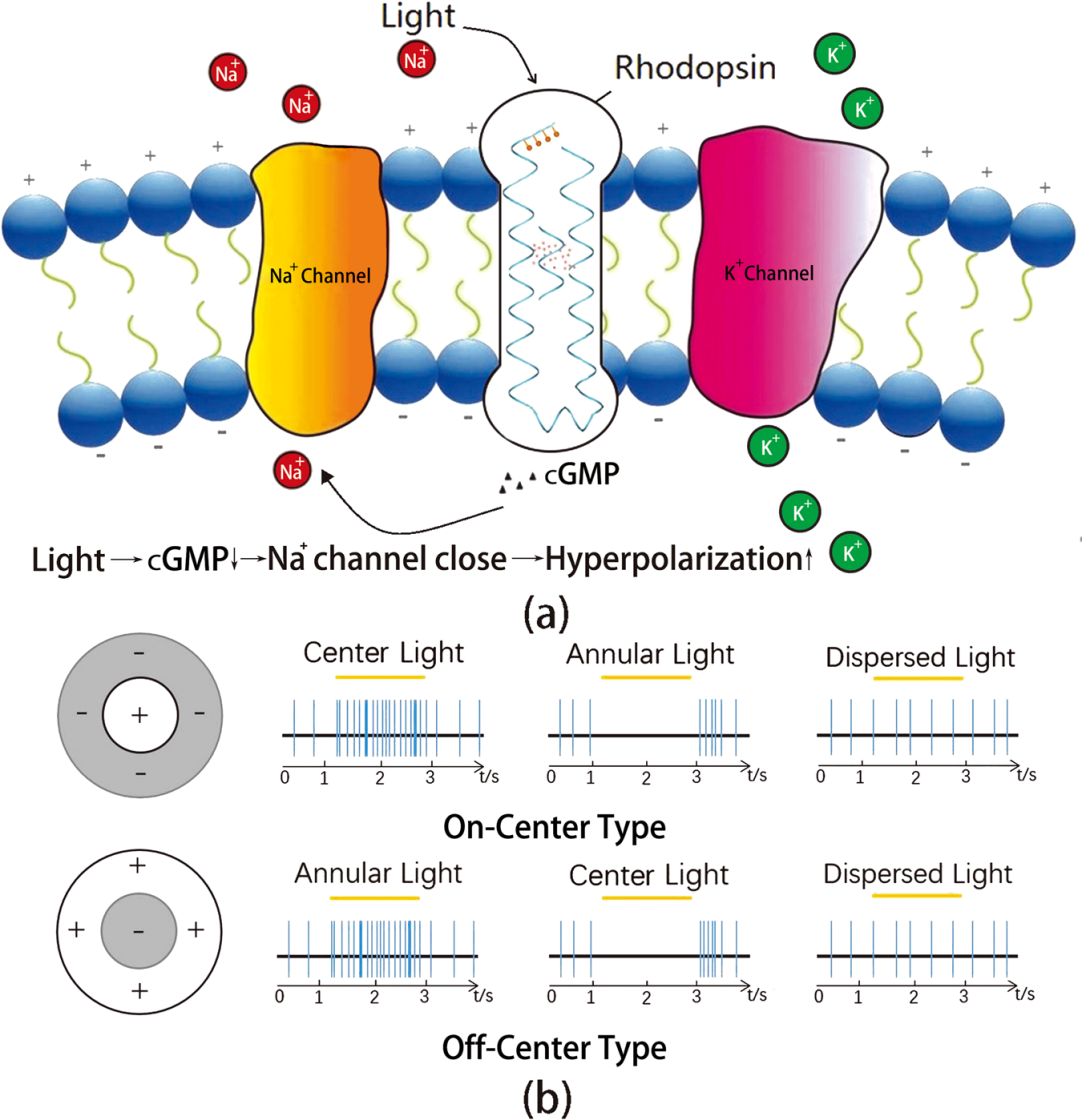
(**a**) Light changes the membrane potential of rod cells in retina. Light is absorbed by pigments (include opsin and retinal) which located in the rhodopsin. Photon will induce the isomerization of retinal and the concentration of cGMP (a kind of intracellular messenger) will decrease. The activity of Na^+^ channels has a positive correlation with the concentration of cGMP. So the light will hyperpolarize the cell membrane and the spiking frequency of rod cells will decrease. (**b**) The on-center receptive field and off-center receptive field of ganglion cells (GCs). The yellow bar represents the specific illumination period and the blue bars represent the spikes which recorded from the neuron. For the on-center cells, center illumination will increase the spiking frequency and the surrounding illumination will decrease the spiking frequency. For off-center cells, by contrast, the surrounding illumination will increase the spiking frequency and the center illumination will decrease the spiking frequency. For two types of cells, the dispersed light makes no difference to their spiking frequency. In summary, the GCs are sensitive to the difference of light rather than the light itself. By this feature, GCs can extract the shape of objects and code them into spike trains.

There are two types of photoreceptor cells in the retina: cone cells and rod cells^3,4^. Photon is absorbed by pigments (include opsin and retinal) which located in the rhodopsin **(**Fig. 1. (a)**)**^5,6^. Photon will induce the isomerization of retinal so that the concentration of cGMP (a kind of intracellular messenger) will decrease^7–10^. The decreasing concentration of cGMP will close the Na^+^ channels so that the inward sodium current will decrease and the cell membrane will be hyperpolarized. So the membrane potential of photoreceptor cell has a positive correlation with the concentration of cGMP. Because of illumination will decrease the concentration of cGMP, it is harder for photoreceptor cells to create spikes when they are exposed in light. When there is almost no cGMP in the cell, cell won’t fire. In conclusion, the illumination will increase the equivalent threshold (V_th_) of photoreceptor cells. The spiking frequency of cells will decrease when the illumination is increased.

The photoreceptor cells code the information into a spike train and send it to the ganglion cells (GCs). Experiment results show that the GCs in the retina of monkey and cat have two types of receptive fields: the on-center type and the off-center type (Fig. 1. (b))^11^. In Fig. 1. (b), the yellow bar represents the specific illumination period and the blue bars represent the spikes which recorded from the neuron. The on-center cells are excited (i.e. release more spikes) when the center of receptive field is illuminated. But when surrounding areas are illuminated, the excitation will be suppressed and the cell won’t release spike. On the contrary, center-illumination will suppress the off-center cells and surrounding illumination will excite them. For two types of cells, the dispersed light makes no difference to their spiking frequency. In summary, GCs are sensitive to the difference of light rather than the light itself^12^. By this way, the shape of objects can be extracted and sent to higher nerve centers. In the higher level of visual perception processes, more complicated spatial features can be extracted. By these hierarchical perception processes, the vision can be formed in the brain.

In order to construct artificial shape perception retina network, two challenges need to be overcomed. In general, information is represented by electrical signals and delivered by synapses between neurons. Conventional integrated-and-fire neuron can be used to complete data processing. However, in photoreceptor neurons, membrane potential is not only affected by other neurons but also the light signals in the environment. The conventional integrate-and-fire model cannot completely describe the photoreceptor cells. So, on the cell level, a tunable artificial neuron needs to be constructed in order to mimic photoreceptor cells and ganglion cells. As mentioned above, the receptive field of ganglion cell is the core of shape perception process. So, on the network level, in order to realize the receptive field of ganglion cell, the connections between photoreceptors and ganglion cell need to be constructed.

In traditional CMOS based electrical neurons, the cell membrane was usually mimicked by a capacitor^13–15^. However, even in the most advanced technology node, realizing the capacitance densities of biological membranes (~10fF μm^−2^) is still a challenge^16^. Researchers from IBM have put forward a concept that the voltage between two terminals of phase-change RAM (PCRAM) can be used to mimic the membrane potential^17^. With this concept, they built the integrate-and-fire neurons, and solved the integration problem of electronic neurons. However, the modulation effects during integrate period were still not included in their model. The one-transistor-one-memristor (1T1M) structure which formed by one memristor and one MOSFET in serial is widely used in resistive memories and neuromorphic systems^18–23^. Because of the memristor can be integrated on the drain electrode of MOSFET, the 1T1M device can achieve a high density of integration^24^. Setting a leading-out terminal between memristor and MOSFET, then the 1T1M structure can be seen as a four-terminals device, and it can conveniently be designed to mimic the modulation effects of light signals in the environment. In this paper, a novel tunable integrate-and-fire memristive neuron was built on 1T1M structure. With these neurons, we mimicked the behaviour of photoreceptor cells and demonstrated the concept of receptive field of ganglion cells (GCs). In the end, an artificial shape perception retina network was built. The simulation results show that the artificial shape perception retina network can extract shape information from the image and transform it into spike frequency realizing the function of computing in sensor.

### Tunable Memristive Neuron

**Figure 2:**
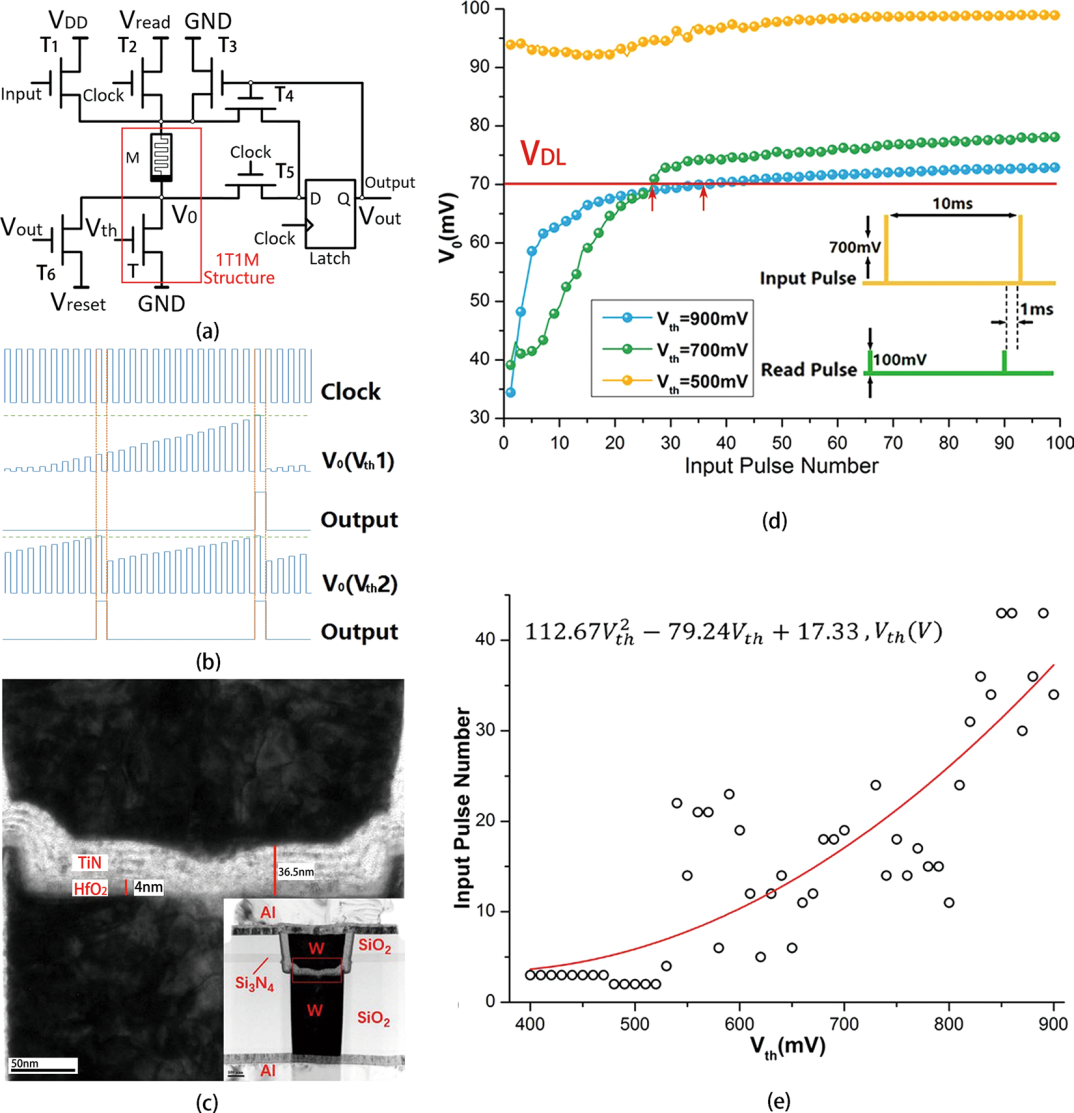
(**a**) The structure of tunable memristive neuron. When there is an input pulse, the resistance of memristor will decrease. V_read_ is divided by memristor and MOSFET and V_0_ is the voltage between two terminals of MOSFET. When there is a rising edge of read clock, the D-Latch will read the V_0_. If V_0_ reaches the threshold of D-Latch (V_DL_), the output of D-Latch will change to a high level. Then the reset circuit will work. When the falling edge of the read clock is coming, the memristor and the output of D-Latch will be reset. (**b**) The sequence diagram of memristive neuron. (**c**) The TEM image of the HfO_x_-based memristor. (**d**) The relationship between V_0_ and input pulse number under different gate potentials. The higher gate potential, the harder for V_0_ to reach V_DL_. (**e**) The relationship between the gate potential (V_th_) and input pulse number when there is one output spike. When the V_th_ lower than 400 mV, the relationship will nearly be a constant, we deem that three input pulses can trigger an output spike.

In general, the behavior of neurons can be described by integrate-and–fire model. However, as mentioned above, for photoreceptor cells, their behaviors are not only affected by synaptic inputs but also the light signals in the environment. So, the conventional integrate-and-fire model cannot completely describe these neurons. By using 1T1M structure, the changes of membrane potential resulting from synaptic inputs and light signals can be separated. The voltage changes between two terminals of memristor can mimic the changes of membrane potential caused by synaptic inputs. The voltage changes between two terminals of MOSFET can mimic the changes of membrane potential caused by light signals in the environment. With 1T1M structure, the tunable integrate process of photoreceptors can be described. By means of peripheral circuit, the neuron can realize the fire process and reset process (Fig. 2. (a)). In this work, the function of peripheral circuit is realized by Agilent B1500A (See Supplementary Information, Fig. S1. (b)).

In Fig. 2. (a), V_0_ represents the membrane potential. The gate potential of MOSFET (V_th_) is corresponding to equivalent threshold of photoreceptor cell. As mentioned in introduction, there is a positive correlation between the light intensity and the equivalent threshold of photoreceptor cell. So, in the following analysis, V_th_ is used to represent both the equivalent threshold of photoreceptor cells and the light intensity. For a specific light intensity, the gate potential is a constant so that the MOSFET can be seen as a resistor. When there is an input pulse, T_1_ will be turned on. As a consequence, the resistance of memristor will decrease and V_0_ will increase. In fact, if the gate potential is set to be a constant, the artificial neuron will work in traditional integrate-and-fire mode and can be used to mimic universal neurons. T_2_, T_5_ and D-Latch are used to read the membrane potential V_0_. When there is a rising edge of read clock signal, if V_0_ reaches threshold of the D-Latch (V_DL_), the D-Latch will latch a high level. At the same time the reset circuit will be activated. T_3_, T_4_ and T_6_ will be turned on and V_0_ is codetermined by the resistance of all MOSFETs. At this time, the circuit is in a transition stage and the value of V_0_ has no effect on output. When the falling edge of read clock is coming, T_2_ and T_5_ will be turned off so that the memristor will be reset by T_6_ and T_3_. At same time, the input of D-Latch will be reset to low level by T_3_ and T_4_. The output of D-Latch will come back to low level when the next rising edge of read clock is coming. By this way, the memristive neuron finished the process of integrate-and-fire. It is a remarkable fact that the spiking frequency of memristive neuron has an upper limit which determined by the read clock. In fact, the highest spiking frequency of the memristive neuron equals to the half of the read clock frequency. Moreover, the rising edge of the output spike is coincided with the rising edge of read clock. These features are determined by the circuit structure, and will be used to construct the artificial retina network.

In the photoreceptor cells, the more illumination, the lower concentration of cGMP. As a consequence, the outward sodium current will be larger than inward sodium current and the membrane potential will decrease. There is a corresponding process in the memristive neuron. The increasing of V_th_ will increase the drain current of MOSFET (i.e. the outward sodium current of neuron) and will cause the decrease of V_0_, so that the neuron needs more input spikes to trigger an output spike. Figure 2. (b) is a schematic diagram which shows the differences of integrate rate when applying different gate potentials (V_th1_, V_th2_, V_th1_ > V_th2_). The higher gate potential, the slower integrate rate. So the gate potential will affect the spiking frequency of memristive neuron. Figure 2. (d) shows the result of a measurement and it is consistent with Fig. 2. (b). Under the low V_th_ (500 mV), V_0_ has exceeded the V_DL_ even though there isn’t an input pulse. In this case, the spiking frequency of memristive neuron equals to half of the read clock frequency. The drain current is very small when the gate voltage is equal to 500 mV, so the resistance change rate of the memristor and the increase rate of V_0_ are very slow. Besides, because of the equivalent resistance of MOSFET is much higher than the memristor (See Supplementary Information, Fig. S2. (c)), V_0_ has a smaller raise. Under the middle V_th_ (700 mV, can be seen as V_th2_), the drain current has increased and the increase of V_0_ has been accelerated. At this time, the resistance of memristor and MOSFET are in the same level, so the increase of V_0_ is more obvious. Because of the decrease of MOSFET equivalent resistance, the initial state of V_0_ is below the V_DL_, and the memristive neuron shows the function of “integrate-and-fire”. Under the high V_th_ (900 mV, can be seen as V_th1_), there is a further increase of drain current, and the V_0_ shows a sharp rising edge. Meanwhile, because of the equivalent resistance of MOSFET is very small, the initial state of V_0_ has a further decrease. It is easy to see from the Fig. 2. (d) that the higher gate potential, the longer time for V_0_ to reach the V_DL_ and the larger spiking interval of memristive neuron. By the tuning effect of MOSFET, the memristive neuron can response to spike input and analog signal at the same time, which is the key point in mimicking the photoreceptor cells. Figure 2. (e) shows the number of pulses required to fire the neuron under different V_th_. Obviously, with the increase of the V_th_, neuron needs more input pulses to trigger an output spike. When the V_th_ lower than 400 mV, the relationship will nearly be a constant, we deem that three input pulses can trigger an output spike.

### Artificial Shape Perception Retina Network

Organisms have a hierarchical perception process. Retina plays an important role in the formation of vision. As the visual receptor, the absorption of light and the pretreating of light signals are finished in retina. Retina needs to attract useful information from the light signal and codes it into action potential trains. The ganglion cells (GCs) which located in retina have the function of extracting the edges of image^17^. So the imitation of GCs is the core process of constructing artificial retina. As mentioned in introduction, there are two kinds of GCs. The common characteristic of these GCs is they can compare the output signals of some photoreceptors and they will be excited when there are differences between these signals. So the GC-photoreceptors system can be abstracted as a multi-inputs comparator. This is the core idea of our network designing.

As there is not full knowledge about the representation of information in neural system yet, we made some assumptions in the construction of artificial retina network to simplify circuit designing. First of all, it is observed by biologists that the amplitudes of action potentials are nearly equal^17,25^. So we can assume that the electrical signals in neural systems only have two values: the high level and the low level (i.e. the “1” and the “0”). The “1” represents that there is an action potential at the moment, and the “0” represents that there isn’t an action potential at the moment. Possibility of the emergence of action potential (i.e. high level) is adjusted by the neuron. Secondly, the neuron has a silent period which named absolute refractory period^17,25–27^. During this period, the neuron cannot be stimulated to fire. Obviously, the spiking frequency of neuron is limited by the absolute refractory period. So, it is reasonable to assume that there is a local clock signal in neural system. The frequency of this local clock (f_0_) can be described by the following formula:

$${f}_{0}=\frac{1}{spkie\,period+refractory\,period}$$

and the spiking frequency of all neurons in the local area will not be higher than f_0_. As mentioned above, the highest spiking frequency of memristive neuron equals to the half of read clock frequency. So the read clock can be used as local clock in the network. Thirdly, in this work, the spiking frequency of neuron is regarded as the representation of information in neural system. As the arrival instants of spikes aren’t involved in this representation, so the artificial retina network in our work is different with conventional Spiking Neural Network (SNN)^28^. The last assumption is that an integrate-and-fire neuron can be regarded as a frequency divider which has some degrees of random. In this work, because of the integrate-and-fire process of memristive neurons is affected by physical signals (light signals) or chemical signals (neural messager proteins) rather than only affected by other neurons, the memristive neurons can be abstracted as random frequency dividers which modulated by the outside signals. In Fig. 2. (e), we have showed the correlation between the gate potential (V_th_) and input pulse number when there is one output spike. This correlation can be also well represented in the model we built for this tunable memristive neuron (See Supplementary Information Fig. S6) and based on this model, the artificial shape perception retina network was constructed.

**Figure 3:**
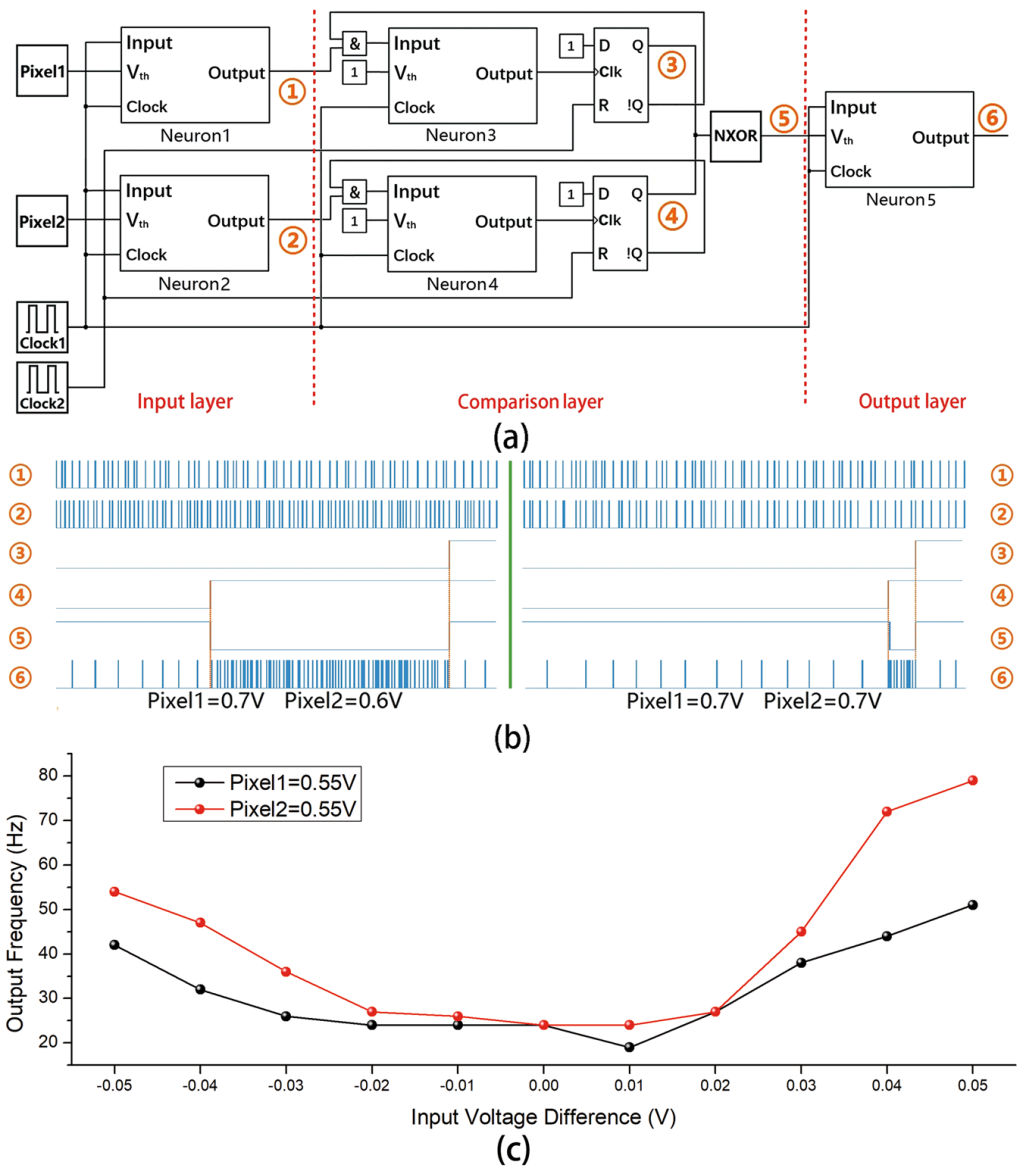
(**a**) The structure of double-inputs comparator. The circuit consists of three layers: input layer, comparison layer and output layer. (**b**) The waveform of six key nodes in the network under different inputs. The more difference between two input signals, The higher frequency of output. (**c**) The correlation between output frequency and input voltage difference. Black line (red line) represents the correlation between output frequency and voltage difference when Pixel1 (Pixel2) voltage is kept at 0.55 V.

Figure 3. (a) shows the structure of double-inputs comparator of detecting illumination differences. The model of memristive neuron which used in this network was fitted by experimental data. There are six key nodes in the network, and Fig. 3. (b) shows the waveform of these notes. Double-inputs comparator can be divided into three layers: the input layer, the comparison layer and the output layer. The input layer consists of Neuron1 and Neuron2. The V_th_ terminals of two neurons are directly connected with analog inputs from the outside (i.e. the grayscale of two pixels. In the simulation, pixel1 and pixel2 are double floating point values between 0 V and 1 V.). Clock1 is the local clock which has the highest frequency in the network. The local clock signal is divided by Neuron1 and Neuron2 under the modulation of pxiel1 and pixel2. It can be seen from Fig. 3. (b) ① and ②, the increase of V_th_ will induce the decrease of the spiking frequency. This feature is determined by the structure of memristive neuron and is consistant with photoreceptor cells. Obviously, the input layer has coded the grayscale of image (the grayscale information can come from sensor or storage device) into the frequency of spike trains.

The main function of comparison layer is to obtain the difference of two input frequencies. The comparison layer mainly consists of two neurons (Neuron3 and Neuron4), two D-Latches and a XNOR gate. As the function of comparison layer is only related to the pulse input, the V_th_ is set to be a constant potential (1 V) and the neurons work in traditonal integrate-and-fire mode. Clock2 is the reset clock of D-Latch. In a period of Clock2, the Neuron3 and Neuron4 only fire one time, and the higher frequency of input, the shorter time before neuron to fire. By this way, the fire moment can represent the frequency of input. The D-Latch transforms the pulse signal into the rising edge signal and sends them to the XNOR gate forming an output. In Fig. 3. (b), ③④⑤show the waveforms of three key nodes in the comparison layer. Obviously, the earlier arrival rising edge is corresponding to the higher frequency of input. The larger difference of two frequencies, the larger time difference of two rising edges. The comparison layer codes the difference of two input frequencies into the duration of low level of output.

The level signal generated in the comparison layer will be transformed into spike trains in the output layer. Because of the function of output layer is only related to the level signal, the pulse input terminal is connected to the local clock. From ⑤ and ⑥ in Fig. 3. (b), it can be seen that the longer duration of low level of the input, the more spikes will be generated in one period of Clock2, and the higher spiking frequency of the output layer. Figure 3. (c) shows the correlation between output frequency and input voltage difference of double-inputs comparator (for 3D-graph, see Supplementary Information Fig. S4). In conclusion, the double-inputs comparator codes the difference of two analog inputs into a spike train, and the frequency of output has a positive correlation with the difference of two inputs.

**Figure 4:**
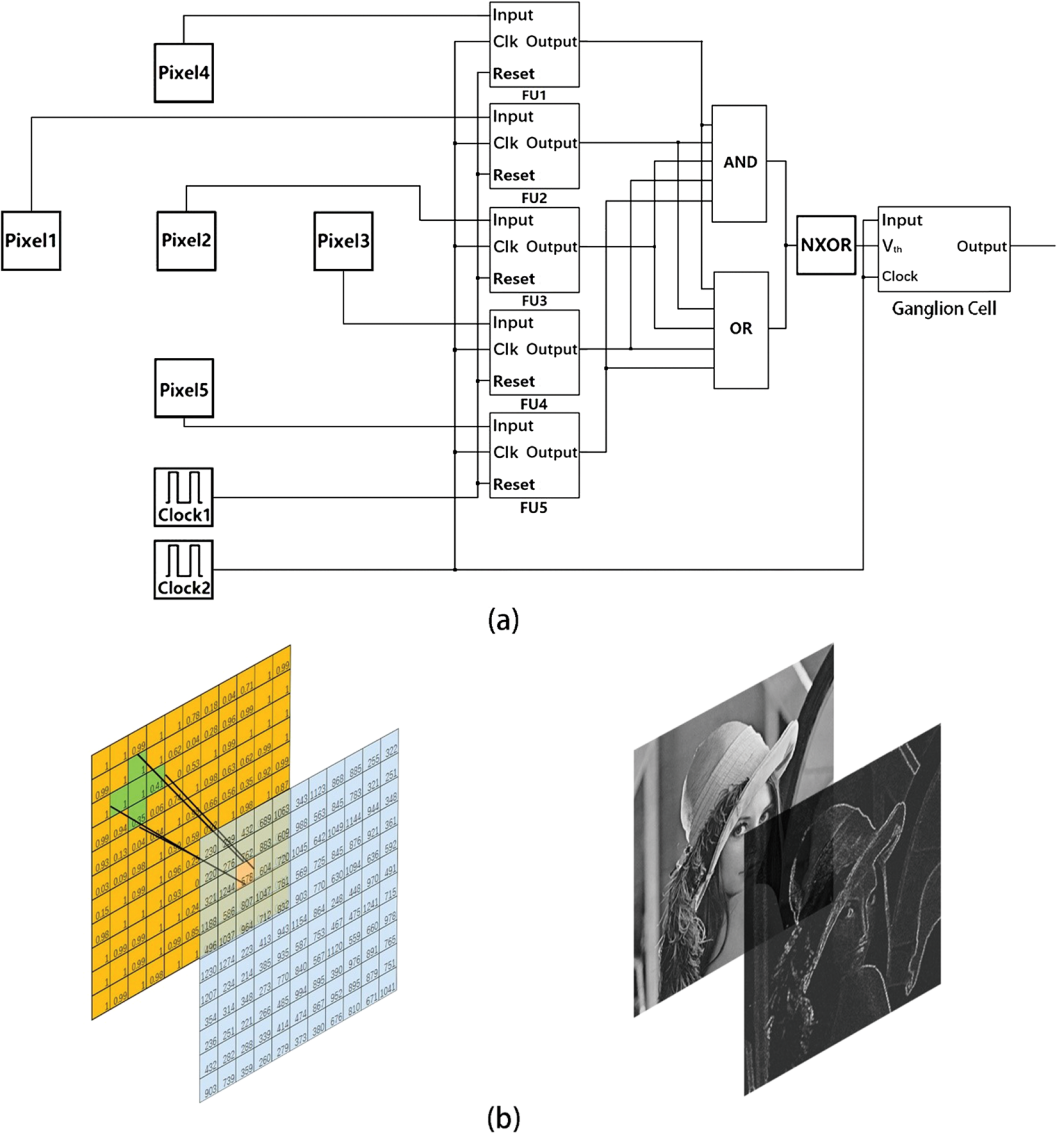
(**a**) The structure of five-inputs comparator which is the unit circuit of the artificial retina network. (**b**) With artificial shape perception retina network, the grayscale matrix is transformed into the frequency matrix, and the edges in the image were detected (the image used for processing comes from^27^).

One of the merits of this double-inputs comparator is that it can be expanded to the multiple-inputs comparator. For example, a five-inputs comparator can be built in the same approach and be applied to mimic the receptive field of the ganglion cells (GCs) in retina. Figure 4. (a) shows the structure of five-inputs comparator. In this structure, the function units (FUs) consists of one input layer and one comparison layer which structures are same with what have shown in the Fig. 3. (a). The OR gate and the AND gate extract the first arrival rising edge and the last arrival rising edge from the outputs of FUs. The time difference between two rising edge determines the spiking frequency of GC. Five-inputs comparator with the center-surround receptive field is the unit circuit of artificial shape perception retina network, and can extract the shape information from images. The shape extraction process is illustrated in Fig. 4 (b)(the image used for processing comes from^29^). With artificial shape perception retina network, the grayscale matrix is transformed into the frequency matrix, and the edges in the image were detected. Because of there is a parallel correlation between every unit circuit, the artificial shape perception retina network has much higher efficacy than traditional CPU-based serial structures.

## Discussion

The variation of switching parameters is one of the major challenges to birth the memristive computing system. It can be seen from the Fig. 1. (e), the dispersed points show there has a distribution of integrate parameters of memristive neuron which caused by the variation of the memristor (Supplementary Information Fig. S5 shows the histogram of deviation between fitting curve and experimental data). Considering this variability of memristor, in the model of memristive neuron, a random number is used to correct the output frequency (See Supplementary Information Fig. S6 (a)). However, in fact, these variations have little impact on our network. It is because the special frequency-based information representation has an average effect so that the variation will be suppressed at some degrees. A little bit more spikes or fewer spikes won’t destroy too much information coded in the spike frequency. Moreover, because of the two-value representation of action potentials (i.e. the network can be seen as a digital circuit at some degrees), the variation of spike amplitude has little impact on information integrity. By these robust information coding principles, the nervous systems of organisms can work in many hostile environments and these principles are exactly what we need in circuit designing.

There are several artificial neurons have been put forward in recent years, for example, the neuristor which based on Mott memristor and phase change neuron which based on PCRAM. In the neuristors or traditional CMOS-based neurons, the membrane potential is represented by the voltage between two terminals of a capacitor. However, it is hard to fabricate large bulk capacitors on silicon substrate. As a consequence, the integrate ability of capacitor-based artificial neurons is limited by the capacitance (i.e. a small number of input spikes will trigger an output). In the phase change neuron, the capacitor has been replaced by a PCRAM, so a better integrate process has been achieved. This strategy has been emulated in our work and a memristor based 1T1M structure has been used to control membrane potential. The greatest beneficial of our model is that the influences of environment (both physical and chemical) can be introduced in the integrate process. These influences can be very important in information processing. For example, only the photoreceptor cells are able to be influenced by light signals can the vision be realized. However, there are still some weakness points of our neuron model. Because of there needs to be a reset circuit, the complexity of this neuron model is hard to be reduced. Replacing bipolar devices by unipolar devices may simplify the reset circuit and achieve a higher integration level. For designing an artificial neuron which is full-featured and simple-structured, a further work still needs to be done in the future.

## Conclusion

Machine vision is one center of attention in the field of information science. As an important neural unit, retina works with a high efficiency data processing strategy called computing in sensor. In the neuromorphic circuits, with the lack of suitable electronic neuron elements, the development of artificial retina circuit has been limited. In this paper, we proposed a new type of memristive neuron based on one-transistor-one-memristor (1T1M) structure which can integrate spike signal and analog signal at same time. The memristor and MOSFET integrate spike signal and light signal respectively, and the fire process can be realized by peripheral circuit. With these neurons, we demonstrated the concept of receptive field of ganglion cells (GCs), and the artificial shape perception retina network was built. With the benefit of parallel structure, artificial shape perception retina network has high efficiency on image processing, showing the potential in the mobile terminal applications. Moreover, the information in the network is represented by the frequency of two-value spike trains, so the artificial retina network has a good characteristics of anti-disturbance. Combined with artificial synapses formed by memristors^30–34^, this artificial shape perception retina network can be used as the receptor and is promising for implementing the advanced neuromorphic systems with complicated visual functions.

## Methods

The tunable memristive neuron used in this work is based on a hybrid integration of a HfO_x_-based memristor and a nMOSFET by a standard CMOS process (Fig. 2. (c)). The W/HfO_x_/Ti sandwich structure and MOSFET were integrated by 0.13μm logic process. A tungsten plug was fabricated on the drain electrode of MOSFET and it was used as the bottom electrode of memristor. Then a 5 nm HfO_x_ film was deposited by ALD. At the last, a 10 nm Ti film was deposited on the HfO_x_ by PVD and used as the top electrode. The size of device is 0.26 μm × 0.26 μm, defined by the tungsten plug. See Supplementary Information for more characteristics of the device.

The 1T1M device was characterized by Agilent B1500A (See Supplementary Information Fig. S1). The amplitude and the period of input pulses are 700 mV and 10 ms.The read pulse was a spike train with 100 mV for amplitude and 10 ms for period. Time difference between input signal and read signal is 1 ms. The threshold of cell membrane is set to be 70 mV. The electrical signals used in experiment are generated by Agilent B1500A. The experimental data was used to construct tunable memristive neuron model and the artificial retina circuit was based on this model.

The construction and simulation of artificial shape perception retina network were finished by MATLAB-SIMULINK R2016B. See Supplementary Information Fig. S6 for the circuit diagram.

## Electronic supplementary material

Supplementary Information

## References

[1] Pickett MD, Medeirosribeiro G, Williams RS (2013). A scalable neuristor built with Mott memristors. Nature Materials.

[2] Yumin Kim. (2018). Nociceptive Memristor. Advanced Materials.

[3] Balasubramanian V, Sterling P (2009). Receptive fields and functional architecture in the retina. J. Physiol..

[4] Masland RH (2001). The fundamental plan of the retina. Nature Neuroscience.

[5] Brown PK, Wald G (1963). Visual Pigments in Human and Monkey Retinas. Nature.

[6] Marks WB, Dobelle WH, Macnichol EF (1964). Visual Pigments of Single Primate Cones. Science.

[7] Matthews RG, Hubbard R, Brown PK, Wald G (1963). Tautomeric Forms of Metarhodopsin. Journal of General Physiology.

[8] Fesenko EE, Kolesnikov SS, Lyubarsky AL (1985). Induction by cyclic GMP of cationic conductance in plasma membrane of retinal rod outer segment. Nature.

[9] Stryer L, Bourne HR (1986). G proteins: a family of signal transducers. Annual Review of Cell Biology.

[10] Yau KW, Nakatani K (1984). Cation selectivity of light-sensitive conductance in retinal rods. Nature.

[11] Kuffler SW (1953). Discharge patterns and functional organization of mammalian retina. Journal of Neurophysiology.

[12] Kuffler SW, Nicholls JG (1976). From neuron to brain. Quarterly Review of Biology.

[13] Indiveri G, Chicca E, Douglas R (2006). A VLSI array of low-power spiking neurons and bistable synapses with spike-timing dependent plasticity. IEEE Trans. Neural Networks.

[14] Schemmel J, Fieres J, Meier K (2008). Proc. Wafer-scale integration of analog neural networks. Int. Joint Conf. Neural Networks.

[15] Indiveri G (2011). Neuromorphic silicon neuron circuits. Front. Neurosci..

[16] Gentet LJ, Stuart GJ, Clements JD (2000). Direct Measurement of Specific Membrane Capacitance in Neurons. Biophys. J..

[17] Tuma T, Pantazi A, Le GM, Sebastian A, Eleftheriou E (2016). Stochastic phase-change neurons. Nature Nanotechnology.

[18] Wu MC, Lin YW, Jang WY, Lin CH (2011). Low-Power and Highly Reliable Multilevel Operation in 1T1R RRAM. IEEE Electron Device Letters.

[19] Zangeneh M, Joshi A (2014). Design and Optimization of Nonvolatile Multibit 1T1R Resistive RAM. IEEE Transactions on Very Large Scale Integration Systems.

[20] Chen YY, Komura M, Degraeve R, Govoreanu B (2013). Improvement of data retention in HfO_2_/Hf 1T1R RRAM cell under low operating current. Electron Devices Meeting.

[21] Zhuo-Rui Wang. (2017). Functionally Complete Boolean Logic in 1T1R Resistive Random Access Memory. IEEE Electron Device Letters.

[22] Ambrogio S, Balatti S, Nardi F, Facchinetti S, Ielmini D (2013). Spike-timing dependent plasticity in a transistor-selected resistive switching memory. Nanotechnology.

[23] Xue Yang. (2016). Nonassociative learning implementation by a single memristor-based multi-terminal synaptic device. Nanoscale.

[24] Philip Wong. H-S (2012). Metal–Oxide RRAM. Proceedings of the IEEE.

[25] Kandel E. R., Schwartz J. H., Jessell T. M., Siegelbaum S. A. and Hudspeth A. Principles of Neural Science, 5th ed. J. New York, NY: McGraw-Hill., **4**, 1414 (2000).

[26] Gallistel CR, Rolls E, Greene D (1969). Neuron Function Inferred from Behavioral and Electrophysiological Estimates of Refractory Period. Science.

[27] Rolls ET (1971). Absolute refractory period of neurons involved in MFB self-stimulation. Physiology & Behavior.

[28] Ghosh-Dastidar S, Adeli H (2009). Spiking Neural Networks. International Journal of Neural Systems.

[29] Can Li. (2018). Analogue signal and image processing with large memristor crossbars. Nature Electronics.

[30] Yi L (2014). Activity-dependent synaptic plasticity of a chalcogenide electronic synapse for neuromorphic systems. Scientific Reports.

[31] Wang Z (2017). Memristors with diffusive dynamics as synaptic emulators for neuromorphic computing. Nature Materials.

[32] Wang. Z (2018). Fully memristive neural networks for pattern classification with unsupervised learning. Nature Electronics.

[33] Yi Li YZ (2013). Ultrafast Synaptic Events in a Chalcogenide Memristor. Scientific Reports.

[34] Chang T, Jo S-H, Lu W (2011). Short-Term Memory to Long-Term Memory Transition in a Nanoscale Memristor. ACS Nano.

