# Supplementary material for: Artificial Shape Perception Retina Network Based on Tunable Memristive Neurons
Source: Sci Rep. 2018 Sep 13;8:13727. doi: 10.1038/s41598-018-31958-6 (PMC6137125; doi:10.1038/s41598-018-31958-6)
Supplement: Supplementary file 1 — Supplementary Information [file 41598_2018_31958_MOESM1_ESM.docx]

Supplementary Information

Artificial Shape Perception Retina Network Based on Tunable Memristive Neurons

Lin Bao, Jian Kang, Yichen Fang, Zhizhen Yu, Zongwei Wang, Yuchao Yang, Yimao Cai* and Ru Huang*

In the multi-inputs memristive neuron, the 1T1M structure formed by one memristor and one NMOSFET in serial was used to mimic the cell membrane of neuron. **Figure S1. (a)** shows the devise structure of 1T1M. The bottom electrode of HfO_x_-based memristor connects with the drain electrode of NMOSFET by a tungsten plug forming the serial structure. Setting a leading-out terminal between memristor and MOSFET, then the 1T1M structure can be seen as a four-terminals device. Figure S1. (b) shows the characterization process of the device. Keeping the gate potential of MOSFET a constant (for example, 700mV). The pulse generator will apply pulses on the top electrode of memristor so that the resistance of memristor will decrease. By applying the read pulses (100mV), Agilent B1500A can read the intermediate potential (V_0_) and one of the curves in the Figure 2. (d) can be obtained.

**Figure S2** shows the electrical properties of memristor and MOSFET. From Figure S2. (a) and (b), it can be seen that the memristor shows a good gradual conductance tuning characteristic which is important to mimic the integrate process of membrane. Figure S2. (c) shows the relationship between the effective resistance and the gate potential of MOSFET. With the decreases of gate potential, the effective resistance of MOSFET increases dramatically, so that the rising velocity of V_0_ will be slowed and the initial value of V_0_ will increase. This phenomenon has been shown in Figure 2. (d). By tuning the gate potential of MOSFET, the membrane potential affected by physical or chemical signal in the environment can be mimicked.

We performed Energy Dispersive X-ray (EDX) to analyze the specific distribution of each element, as shown in **Figure S3**. Obviously, a clear lateral hierarchical structure can be seen from the DEX, a stack of W/TiN/Ti/HfO_x_/W from the top to end.

**Figure S4** shows the relationship between the output frequency and two input values of double-inputs comparator. It can be seen that when the values of two inputs are equal, the output of comparator keeps in a low frequency (the blue areas in the diagram represent the input ranges which lead to low output frequency).

**Figure S5** shows the histogram of deviation (i.e. the absolute value of N_exp_ - N_fit_) between experimental data and fitting formula (δN) in the Figure 2. (e) and the average deviation is 4.8627. This deviation results from the variation of the memristor and should be considered in the network designing.

**Figure S6. (a)** shows the SIMULINK model of memristive neuron. The Figure 2. (e) has shown the relationship between the gate potential and input pulse number when there is one output spike.

$$Input Pulse Number=\left\{ \begin{aligned} 112.67V_{th}^{2}-79.24V_{th}+17.33 V_{th}\geq0.4V \\ 3 V_{th}<0.4V \end{aligned} \right.$$

The formula means that there is a threshold of the input pulse number which determined by the V_th_. When the number of input pulses reaches the threshold, the circuit will output a pulse. In the model, a Counter calculates the number of input pulses and the Relational Operator finish the comparison process. With the J-K Flip-Flop and the D Latch, the output pulses can synchronize with the read clock. A random number (the mean value is 0 and the variance is 5, these values come from figure S5) was added on the input pulse number in order to describe the random property of the memristive neurons. In summary, the frequency of output signal can be expressed in a formula as following.

$$f_{Output}=\left\{ \begin{aligned} \frac{1}{Input Pulse Number+Random Number} f_{Output}\leq\frac{1}{2}f_{read clock} \\ \frac{1}{2}f_{read clock} f_{Output}>\frac{1}{2}f_{read clock} \end{aligned} \right.$$

From the formula, it can be seen that the memristive neuron has been abstracted as a frequency divider which modulated by the outside signals and the f_Output_ has an up limit which determined by the refractory period of neurons. ^[S1-S3]^

With the SIMULINK model, the five-inputs comparator can be constructed. The input image has been transformed into a grayscale matrix (consists of double floating point values) before the edge detection. By using interface modules, the grayscale matrix can be input into the circuit and the circuit will return an output frequency matrix to the workspace in the MATLAB. With MATLAB, the output matrix was transformed into a new grayscale matrix so that the edge of image can be obtained.

**Reference**

S1. SW Kuffler and JG Nicholls. *Quarterly Review of Biology*, **94**, 303 (1976).

S2. ET Rolls. *Physiology & Behavior*, **7**, 311 (1971).

S3. R Gallistel, E Rolls and D Greene. *Science*, **166**, 1028 (1969)


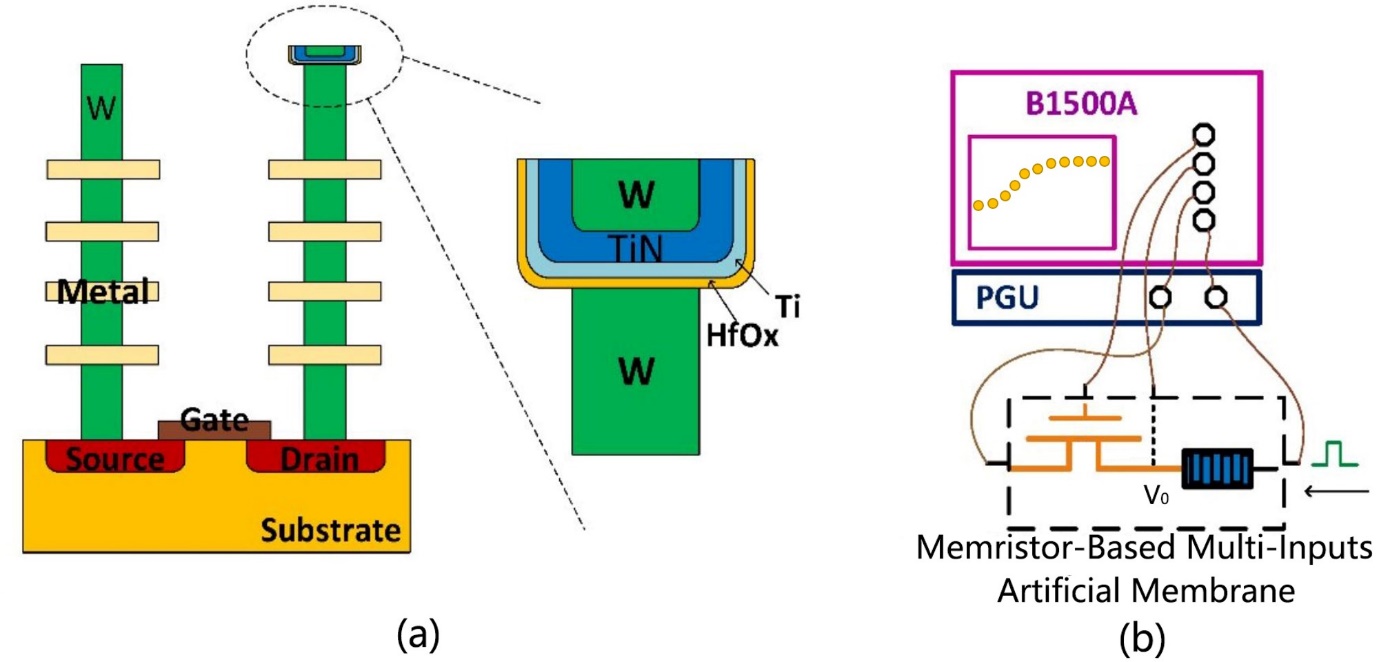


**Figure S1.** Schematic of device structure and measurement. (a) The fabricated device consists of an HfO_x_-based RRAM and a NMOS transistor. (b) The measurement schematic. Agilent B1500A and its pulse generator unite are both used.


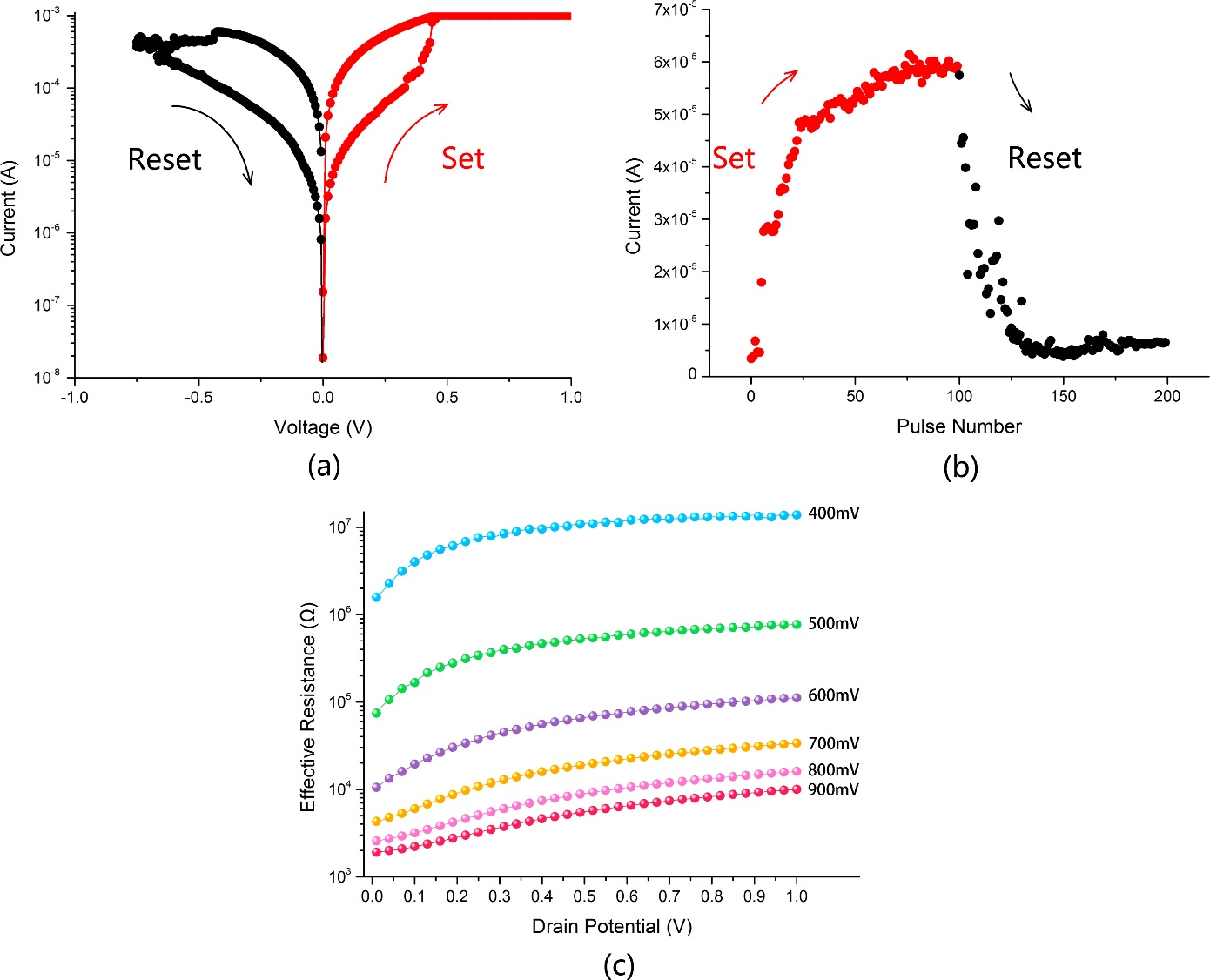
**Figure S2.** The electrical properties of memristor and NMOSFET. (a) The DC characteristic of memristor. (b) The gradual conductance tuning characteristic under continuous positive voltage pulses (700 mV/100 ns, interval = 10 ms) and negative voltage pulses (−1.8 V/100 ns, interval = 10 ms). (c) The effective resistance of MOSFET. Obviously, with the decreases of gate potential, the effective resistance of MOSFET increases dramatically, so that the rising velocity of V_0_ will be slowed and the initial value of V_0_ will increase. By tuning the gate potential of MOSFET, the membrane potential affected by physical or chemical signal in the environment can be mimicked.

**Figure S3**. (a) The EDX (Energy Dispersive X-ray) Line scanning result of HfO_x_-based memristor. (b) The EDX mapping of elements.
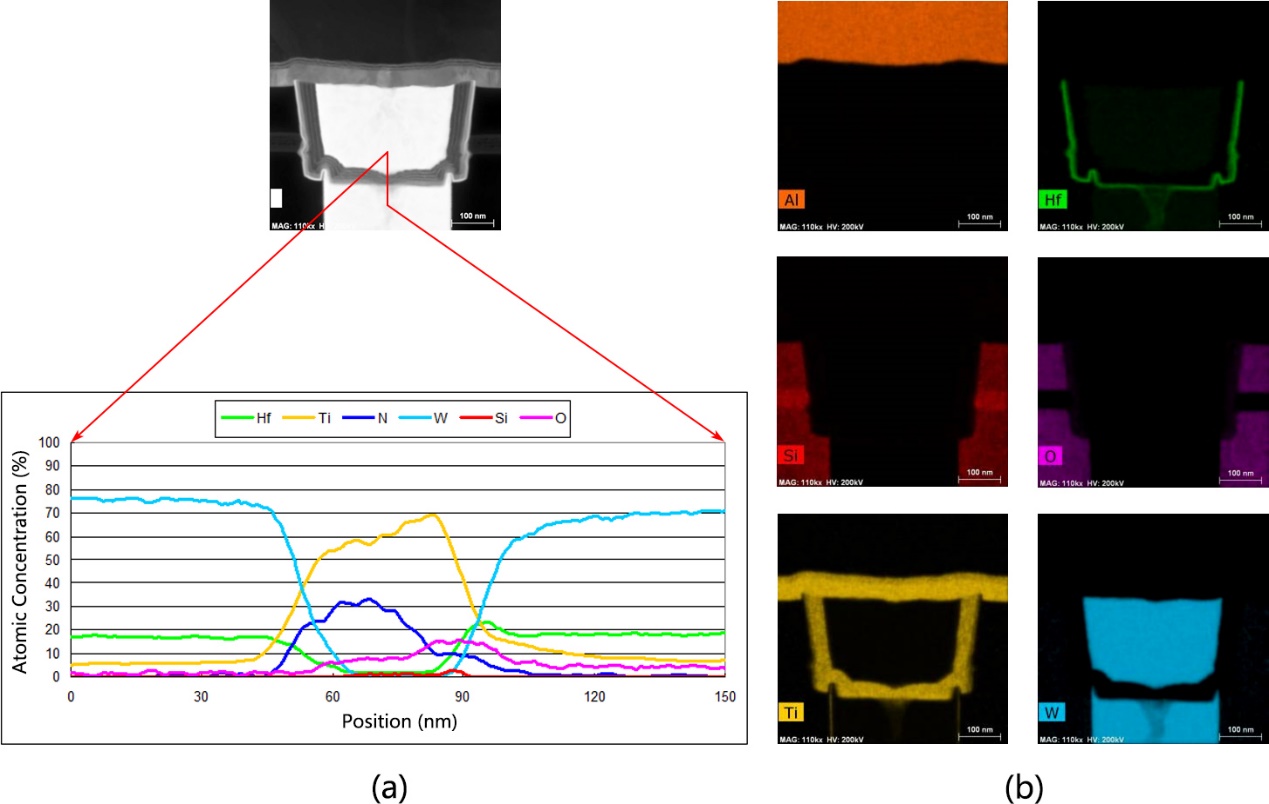


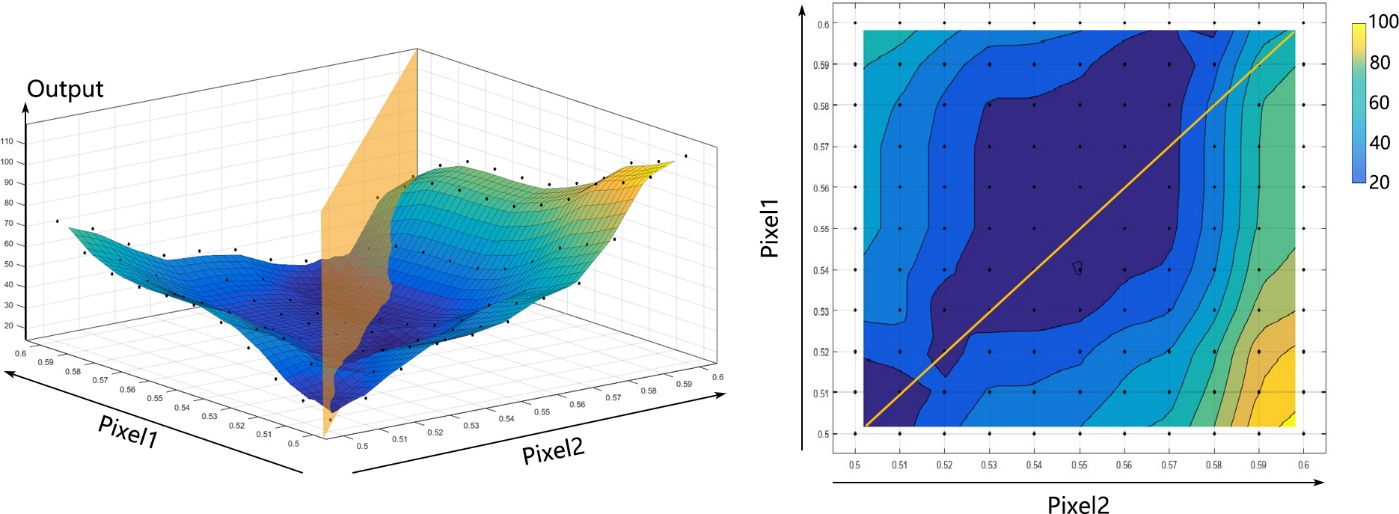


**Figure S4.** The relationship between the output frequency and two inputs of double-inputs comparator. The more difference between two inputs, the higher frequency of output.


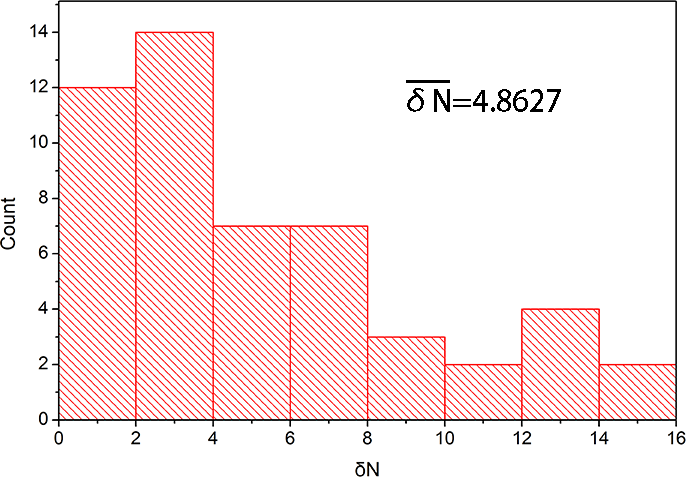


**Figure S5.** The histogram of deviation (i.e. the absolute value of N_exp_ - N_fit_) between experimental data and fitting formula (δN) in the Figure 2. (e). The histogram counted the deviations of different ranges, and the average value of δN is 4.8627.

**
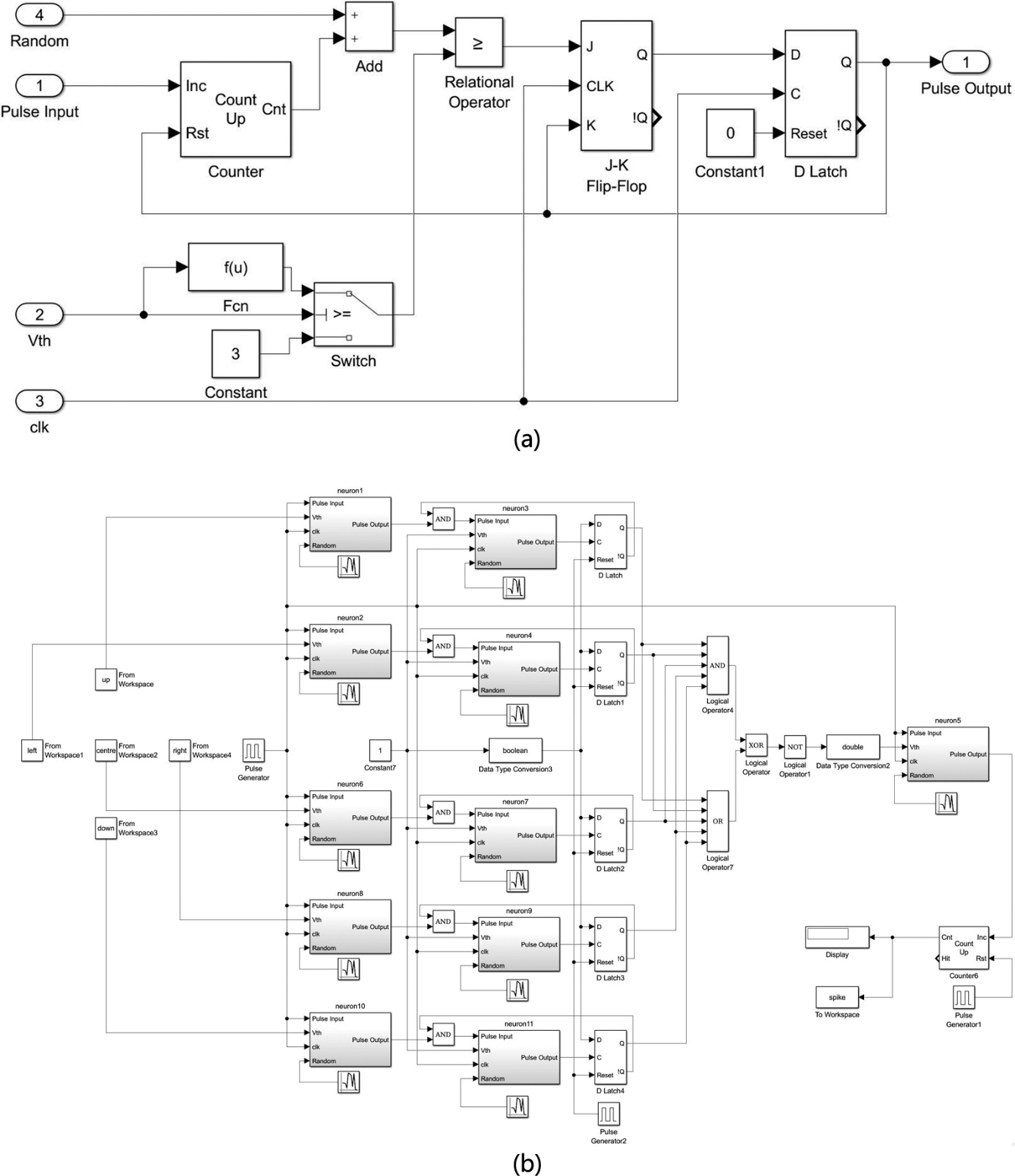
Figure S6**. (a) The SIMULINK model of the memristive neuron. The f(u) is the relationship between V_th_ and the input pulse number which has been shown in Figure2. (e). This relationship comes from the experimental data. In this kind of model, the memristive neuron was regarded as a frequency divider which modulated by the outside signals. (b) The circuit diagram of five-inputs comparator in SIMULINK R2016B software. The input signals come from the workspace in MATLAB and the output frequency is also sent to the workspace.
